# Treatment of pediatric psoriasis with TNF-antagonists: a real-life single-center case series^⋆^

**DOI:** 10.1016/j.abd.2021.10.009

**Published:** 2022-09-05

**Authors:** Matteo Megna, Gabriella Fabbrocini, Lucia Gallo, Angelo Ruggiero, Elisa Camela, Sonia Sofía Ocampo-Garza, Eleonora Cinelli

**Affiliations:** a Section of Dermatology, Department of Clinical Medicine and Surgery, University of Naples Federico II, Naples, NA, Italy; b Universidad Autónoma de Nuevo León, Hospital Universitario “Dr. José Eleuterio González”, Departamento de Dermatología, Monterrey, Nuevo León, México

Dear Editor,

Psoriasis is a chronic inflammatory skin disease.^1^ About 30% of cases include pediatric patients.^1^ It impacts not only children themselves, but also their parents and caregivers, affecting their quality of life.^2^ Most forms are mild and benefit from topical therapy.^3^ However, when unresponsive or more severe, they may require systemic treatments, including phototherapy, conventional (acitretin, methotrexate, and cyclosporine), or biological agents.^4^, ^5^ Biological therapy represents a novel and precious therapeutic option for pediatric patients, although data regarding their efficacy and safety are scant as most of the available clinical trials are run on small study populations and less severe forms of the disease. Hence, the importance to provide information on real-life experiences on pediatric psoriasis under biologicals is not trascurable. Nowadays, anti-Tumour Necrosis Factor (TNF)-α (adalimumab and etanercept) have been approved for children from 4 and 6 years of age, respectively, anti-Interleukin (IL) 12/23 (ustekinumab) for patients from 6 years,^6^, ^7^, ^8^ and anti-IL17 (secukinumab and ixekizumab) in children from 6 years of age.^9^, ^10^ In Italy, only anti-TNF-α agents are currently reimbursable by the National Health Care System, whereas ustekinumab and anti-IL17 are awaiting reimbursement. Herein, we report the real-life experience of reimbursable biologicals for pediatric psoriasis patients referred to the Psoriasis Unit of the University Hospital Federico II, Naples, from September 2018 to September 2020. The inclusion criteria of the retrospective study were: i) Moderate-to-severe plaque psoriasis (defined as PASI > 10, and/or BSA > 10 and/or DLQI > 10) diagnosed at least one year before inclusion; ii) Age < 18 years-old; iii) Wash-out period ≥ 4 weeks for systemic therapies (UV treatment included) and ≥2 weeks for topical ones; iv) Subjects starting biological treatment (adalimumab or etanercept originator or biosimilar).

Treatment was given at a pediatric dosage, based on the patient’s body weight. At baseline: i) Personal and demographic data; ii) Psoriasis duration and localization; iii) Presence of psoriatic arthritis (PsA) and duration; iv) Comorbidities; v) Previous systemic therapies; vi) Psoriasis severity using Psoriasis Area and Severity Index (PASI) and Body Surface Area (BSA); vii) Dermatology Life Quality of Index score (DLQI) in patients and caregivers; viii) Blood tests [blood count, transaminases, creatinine, azotemia, glycemia, erythrocyte sedimentation rate, C-reactive protein, cholesterol and triglyceride levels, protein electrophoresis] were recorded. At each follow-up visit (every 12 weeks), PASI and BSA were evaluated. Moreover, the safety profile was assessed by treatment-emergent AEs, physical examination, and laboratory test monitoring. The Declaration of Helsinki was respected through the whole study and informed consent was obtained and signed by each patient or caregiver before the beginning of the study. Continuous variables were displayed as mean ± standard deviation and categorical variables or as the number and proportion of patients. Unpaired Student’s *t*-test was used to calculate the significance of differences in mean values at the different time points of treatment. A p-value of <0.05 was considered statistically significant. All statistical analyses were performed using GraphPad Prism 4.0 (GraphPad Software Inc., La Jolla, CA, USA). Ten patients were included: 60.0% (n = 6) girls, and 40.0% (n = 4) boys with a mean age of 13.90 ± 4.25 years. They all had plaque psoriasis mean duration of 5.20 ± 3.36 years. None had PsA. One patient presented anemia; no other comorbidities were found. All patients were previously treated with topical agents. Regarding previous systemic treatment, 4 (40.0%) and 2 (20.0%) of them received phototherapy and conventional systemic therapy (acitretin and then cyclosporine, and cyclosporine alone), respectively. The majority (60.0%, n = 6) are currently treated with either originator or biosimilar etanercept (mean duration 32.20 ± 10.35 months), whereas the remaining patients (40.0%, n = 4) with either originator or biosimilar adalimumab (mean duration 12.33 ± 12.09 months). One patient who is currently under etanercept had failed previous treatment with adalimumab for secondary inefficacy (loss of PASI75 response after 12 weeks). Mean PASI improved in both patients treated with adalimumab and etanercept from baseline to week 12, and from week 24 to week 72 stable values were reported for both groups (Table 1). The overall baseline mean PASI was 15.8 ± 5.0 (14.8 ± 6.5 for adalimumab versus 16.4 ± 4.4 for the etanercept group), and it reduced to 2.2 ± 2.7 at week 72 (3.0 ± 5.2 for adalimumab versus 2.0 ± 1.5 for the etanercept group). Although the etanercept group included patients with a higher baseline PASI, a lower mean PASI was reached in the follow-up visits (Fig. 1a). No statistically significant differences were found. Mean BSA showed a similar trend, although from week 24 it increased slightly in both groups (Fig. 1b). The significant improvement of the disease was also reflected by the reduction of the impact on quality of life: DLQI decreased from 18.5 ± 5.5 at baseline to 4.1 ± 3 at week 12, up to 2.5 ± 1.3 at week 72. Regarding AEs, only one patient reported a single episode of epistaxis after the first injection of adalimumab, treated with compression. The patient with pre-existing anemia showed persistence of below threshold hemoglobin (11.0 g/dL), not worsening. In conclusion, our real-life study showed good safety and efficacy of biologics in children and adolescents referring to our center for moderate-to-severe psoriasis. Although the study population is limited, PASI and BSA responses reflect the results reported in clinical trials, as mean PASI decreased from baseline (15.79 ± 5.03) to week 24 (1.74 ± 2.59) under adalimumab and etanercept treatment. The same trend was noticed for mean BSA: 28.50 ± 15.16 (baseline) versus 2.80 ± 3.33 (week 24). Interestingly, both clinical values showed a very slight increase from week 48 (Fig. 1), although no statistically significant difference was reached. Moreover, even if PASI and BSA scores remained stable or slightly increased from week 24, patients and especially caregivers experienced an improvement in DLQI scores. Concerning AEs, our real-life results report a lower incidence of infections than those reported in clinical trials which may be attributed to the different population sizes between clinical trials and everyday practice settings.^11^ Real-life experiences can broaden the study population of pediatric psoriatic patients treated with biologics. Treating effectively and safely psoriasis in children echoes in the familial environment, with benefits extending to their parents.

**Table 1:** Study population characteristics of pediatric psoriasis and results of treatment.

**Patients, n**	10
**Age, years**	13.90 ± 4.25 (range 5–17)
**Sex**	
Female	6/10 (60.0%)
Male	4/10 (40.0%)
**Psoriasis duration, years**	5.20 ± 3.36
**Previous treatments, n (%)**	
Topical therapy	10/10 (100.0%)
NB-UVB phototherapy	4/10 (40.0%)
Cyclosporine	2/10 (20.0%)
Acitretin	1/10 (10.0%)
**PASI**	
Baseline	15.8 ± 5.0
Week 12	3.7 ± 2.5
Week 24	1.7 ± 2.9
Week 48	2.2 ± 2.6
Week 72	2.2 ± 2.7
**Adalimumab group (4 patients, 40%)**	
Baseline	14.8 ± 6.5
Week 12	3.8 ± 3.3
Week 24	2.3 ± 3.9
Week 48	2.7 ± 4.6
Week 72	2.7 ± 4.7
**Etanercept group (6 patients, 60%)**	
Baseline	16.4 ± 4.4
Week 12	3.7 ± 2.1
Week 24	1.4 ± 1.7
Week 48	2.0 ± 2.2
Week 72	2.0 ± 1.5
**BSA**	
Baseline	28.5 ± 15.2
Week 12	5.8 ± 3.6
Week 24	2.8 ± 3.3
Week 48	3.7 ± 4.0
Week 72	3.7 ± 3.4
**Adalimumab group (4 patients, 40%)**	
Baseline	26.0 ± 21.9
Week 12	5.3 ± 3.8
Week 24	3.0 ± 4.2
Week 48	3.0 ± 5.2
Week 72	3.0 ± 5.2
**Etanercept group (6 patients, 60%)**	
Baseline	30.2 ± 10.9
Week 12	6.2 ± 3.8
Week 24	2.7 ± 3.0
Week 48	4.0 ± 3.7
Week 72	4.0 ± 2.6

**Figure 1:**
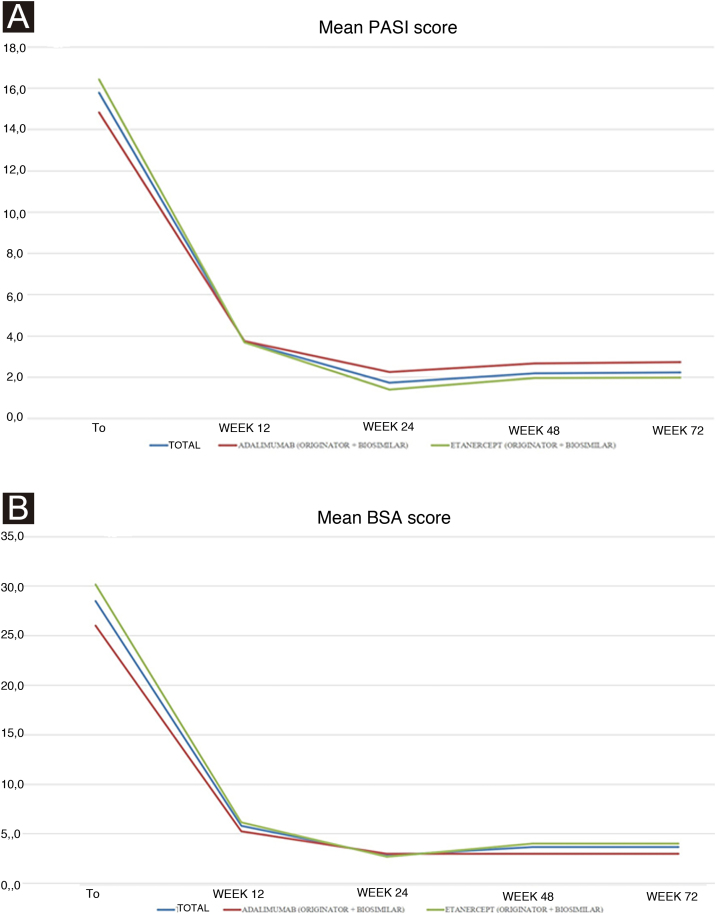
(A) Trends of mean PASI scores in all the study population (blue line), in adalimumab (red line) and in etanercept (green line) group at baseline and follow-ups. (B) Trends of mean BSA scores in all the study population (blue line), in adalimumab (red line) and in etanercept (green line) group at baseline and follow-ups.

## Financial support

None declared.

## Authors’ contributions

All authors contributed to the study's conception and design. Material preparation, data collection and analysis were performed by Matteo Megna, Gabriella Fabbrocini, Lucia Gallo, Angelo Ruggiero, Elisa Camela, Sonia Sofia Ocampo-Garza and Eleonora Cinelli. The first draft of the manuscript was written by Eleonora Cinelli, Gabriella Fabbrocini and Matteo Megna, and all authors commented on previous versions of the manuscript. All authors read and approved the final manuscript.

## Conflict of interest

Doctor Matteo Megna acted as speaker or consultant for Novartis, Eli Lilly and Abbvie. Professor Gabriella Fabbrocini acted as speaker or consultant for Janssen, Leo Pharma, Novartis, Eli Lilly, Abbvie, and Almirall. Doctor Lucia Gallo acted as a speaker or consultant for Lilly, and Pfizer. Doctor Eleonora Cinelli, Doctor Angelo Ruggiero, Doctor Elisa Camela, and Doctor Sonia Sofia Ocampo-Garza have no conflict of interest to declare.
